# 3,3′,4,5′‐Tetramethoxy‐trans‐stilbene and 3,4′,5‐trimethoxy‐trans‐stilbene prevent oxygen–glucose deprivation‐induced injury in brain endothelial cell

**DOI:** 10.1111/jcmm.70008

**Published:** 2024-08-17

**Authors:** Chunxiu Zhou, Yan Zhou, Chi Teng Vong, Haroon Khan, Wai San Cheang

**Affiliations:** ^1^ State Key Laboratory of Quality Research in Chinese Medicine Institute of Chinese Medical Sciences, University of Macau Macau SAR China; ^2^ Macau Centre for Research and Development in Chinese Medicine University of Macau Macau SAR China; ^3^ Department of Pharmacy Abdul Wali Khan University Mardan Mardan Pakistan

**Keywords:** blood–brain barrier, ischemic stroke, oxygen–glucose deprivation, resveratrol derivative, tight junction proteins

## Abstract

Blood–brain barrier (BBB) disruption is a major pathophysiological event of ischemic stroke. Brain microvascular endothelial cells are critical to maintain homeostasis between central nervous system and periphery. Resveratrol protects against ischemic stroke. 3,3′,4,5′‐tetramethoxy‐*trans*‐stilbene (3,3′,4,5′‐TMS) and 3,4′,5‐trimethoxy‐*trans*‐stilbene (3,4′,5‐TMS) are resveratrol derivatives with addition of methoxy groups, showing better pharmacokinetic performance. We aimed to explore their protective effects and underlying mechanisms. Oxygen–glucose deprivation (OGD) model was applied in bEnd.3 cell line, mouse brain microvascular endothelium to mimic ischemia. The cells were pre‐treated with 3,3′,4,5′‐TMS or 3,4′,5‐TMS (1 and 5 μM, 24 h) and then subjected to 2‐h OGD injury. Cell viability, levels of proinflammatory cytokines and reactive oxygen species (ROS), and protein expressions were measured by molecular assays and fluorescence staining. OGD injury triggered cell death, inflammatory responses, ROS production and nuclear factor‐kappa B (NF‐κB) signalling pathway. These impairments were remarkably attenuated by the two stilbenes, 3,3′,4,5′‐TMS and 3,4′,5‐TMS. They also alleviated endothelial barrier injuries through upregulating the expression of tight junction proteins. Moreover, 3,3′,4,5′‐TMS and 3,4′,5‐TMS activated 5′ adenosine monophosphate‐activated protein kinase (AMPK) and endothelial nitric oxide synthase (eNOS). Overall, 3,3′,4,5′‐TMS and 3,4′,5‐TMS exert protective effects against OGD damage through suppressing cell death, inflammatory responses, oxidative stress, as well as BBB disruption on bEnd.3 cells.

## 1 INTRODUCTION

Ischemic stroke, a cerebrovascular disease with high morbidity and mortality, accounts for around 87% of all strokes.^1^ Currently, the only approved therapy by Food and Drug Administration (FDA) for ischemic stroke is recombinant tissue plasminogen activator (tPA) which shows narrow therapeutic index (NTI), potential side‐effect of hemorrhagic transformation, and is treated in very limited patients.^2^ Therefore, there is a significant need to find new effective therapies or drugs to prevent and cure this dangerous disease.

Blood brain barrier (BBB) is considered as an essential gatekeeper to protect the brain through modulating molecule exchange between peripheral blood and cerebral vasculatures.^3^ Generally, brain microvascular endothelial cells (BMECs), pericytes, astrocytes, tight junctions (TJs), adherens junctions, neurons, microglia and basement membrane are all crucial components of BBB.^4^ BMECs are the first line against for neurovascular injury or BBB disruption after ischemia.^5^ In pathologic conditions like acute ischemic stroke, the BBB permeability increases and the BBB disrupts within a few hours, accelerating injury progression, raising the risk of haemorrhage, and even limiting the tPA‐mediated thrombolytic therapy.^3^ Once ischemic stroke happens, insufficient supply of oxygen, glucose and energy, leads to inflammatory responses and oxidative stress and thereby causes degradation of TJ proteins consisting of occludin, claudin‐5 and zonula occludens‐1 (ZO‐1).^6^ During the innate immune response caused by ischemic stroke, the transcription factor, nuclear factor‐kappa B (NF‐κB), is a central regulator essential for numerous genes like cellular adhesion molecules, inflammatory cytokines, transcription factors, immunologic mediators and so on.^7^ Vascular cell adhesion molecule (VCAM)‐1 and intracellular adhesion molecule (ICAM)‐1 are reported as important inflammatory mediators to activate endothelial cells and transmigrate leukocytes during neuroinflammation progression.^2^, ^8^ Besides, increased reactive oxygen species (ROS) contributes to decreased integrity of BMECs and eventual breakdown of BBB. Consequently, even more ROS are produced.^9^, ^10^

Resveratrol, also named as 3,4′,5‐*trans*‐trihydroxystilbene is a well‐known polyphenol, exerting various therapeutic benefits including cerebrovascular‐protective and cardiovascular‐protective effects.^11^, ^12^, ^13^, ^14^, ^15^, ^16^, ^17^ After structural modification of resveratrol, the methoxylated derivatives show higher stability and increased lipophilic properties.^18^ In previous studies, we have illustrated the anti‐inflammatory, anti‐oxidant, anti‐diabetic and vaso‐protective effects of two methoxylated derivatives of resveratrol, 3,3′,4,5′‐tetramethoxy‐*trans*‐stilbene (3,3′,4,5′‐TMS) and 3,4′,5‐trimethoxy‐*trans*‐stilbene (3,4′,5‐TMS).^19^, ^20^, ^21^ Effects of these two derivatives against ischemic stroke are unknown and worthwhile to explore. Oxygen–glucose deprivation (OGD) of BMECs is a well‐established in vitro model to simulate the cellular pathophysiologic conditions of ischemic stroke.^22^ In the present study, we investigated the effects of pre‐treatment of 3,3′,4,5′‐TMS and 3,4′,5‐TMS in immortalized brain capillary endothelial cell line of mouse (bEnd.3) exposed to OGD injury to demonstrate their potential protective effects in ischemic stroke.

## 2 MATERIALS AND METHODS

### 2.1 Cell culture and OGD exposure

**FIGURE 1:**
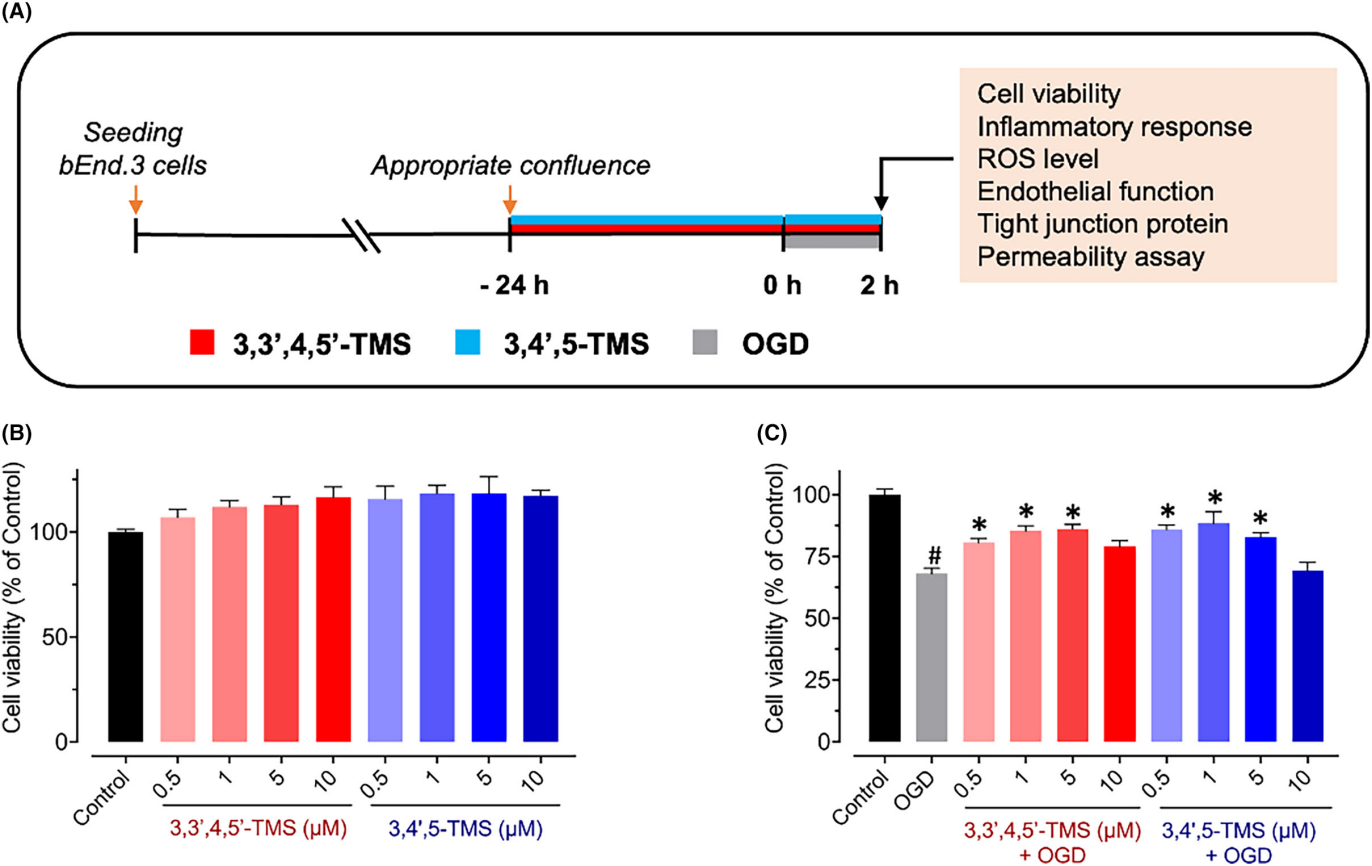
Effects on the cell viability in bEnd.3 cells. (A) Schematic diagram illustrating the experiment protocol to treat bEnd.3 cells with 3,3′,4,5′‐TMS or 3,4′,5‐TMS followed by 2‐h OGD stimulation. (B, C) Cell viability of bEnd.3 cells treated with the two compounds at a range of concentrations (0.5–10 μM) for 24 h with or without 2‐h OGD stimulation. Statistical significance of *p* < 0.05 was indicated with ^#^ (vs. Control) and *p < 0.05 (vs. OGD), *n* = 3.

The source of bEnd.3 cells was America Type Culture Collection (ATCC, USA). Cells were cultured in DMEM (GE Healthcare Life Sciences HyClone Laboratories, USA; Cat#SH30243.01) containing 10% fetal bovine serum (FBS; Gibco, USA; Cat#10270106) and 1% penicillin–streptomycin (P/S; Gibco; Cat#15140122), in incubator with 95% O_2_ and 5% CO_2_ at 37°C. Cells were subjected to 24 h‐pretreatment with 3,3′,4,5′‐TMS (Tokyo Chemical Industry, Japan; purity >98%; Cat#T2842) or 3,4′,5‐TMS (Tokyo Chemical Industry; purity >98%; Cat#T1829) at different concentrations and then to 2 h‐oxygen and glucose deprivation (OGD) at 37°C via a hypoxic chamber (MIC‐101, Billups Rothenberg) after refreshing the culture medium by glucose‐free DMEM (Gibco; #11966025) as shown in Figure 1A. Control cells were cultured in normal DMEM under normal condition. The percentage of oxygen in the chamber was adjusted to less than 0.5%, which was measured by Nuvair O_2_ QuickStick (Nuvair).

### 2.2 Measurement of cell viability

Cell viability of bEnd.3 cells was detected via thiazolyl blue tetrazolium bromide (Sigma Aldrich, USA; MTT; Cat#M6494) assay. In brief, the cells were seeded into 96‐well plates (6 × 10^3^ cells/well, 100 μL medium/well). After adhering overnight, the cells were treated with 3,3′,4,5′‐TMS or 3,4′,5‐TMS at several concentrations of 0, 0.5, 1, 5 and 10 μM for another 24 h. Afterwards, MTT reaction solution (5 mg/mL, 20 μL) was added into each well to be incubated at 37°C for 3 h. Discarding solution in each well carefully, formazan crystals in intact cells were dissolved by the addition of 150 μL DMSO. The absorbance of each well at wavelength 570 nm was finally detected using a SpectraMax M5 multi‐mode microplate reader (Molecular Devices, United States). Meanwhile, the effect of OGD with or without pre‐treatment of 3,3′,4,5′‐TMS or 3,4′,5‐TMS was also measured. The results of cell viability were expressed as % of control group.

### 2.3 Western blotting analysis

The bEnd.3 cells (6 × 10^5^ cells/well, 1.5 mL medium/well in 6‐well plate) were pre‐treated with 3,3′,4,5′‐TMS or 3,4′,5‐TMS (1 and 5 μM, 24 h), and then stimulated with OGD for another 2 h. Finally, the culture media were collected for detection of inflammatory cytokines released and the cells were harvested on ice with RIPA lysis buffer (Beyotime Biotechnology, China; Cat#P0013C) plus 1% phenylmethylsulfonyl (PMSF; Invitrogen, USA; Cat#36978) and 1% protease inhibitor cocktail (Invitrogen; Cat#78427). Upon centrifugation at 15000 rpm for 30 min at 4°C, the supernatants were collected to measure protein contents with BCA assay kit (Beyotime Biotechnology). Protein samples were subjected to SDS‐PAGE and wet transfer onto polyvinylidene fluoride (PVDF) membranes (Millipore, Billerica, MA, USA). After 2 h‐blocking using 5% non‐fat milk or 1% BSA in Tween‐20 phosphate‐buffered saline (TBST) buffer, the membranes were incubated with primary antibodies at 4°C overnight, and with correlated horseradish peroxidase (HRP)‐conjugated secondary antibodies for another 2 h at room temperature. Washing by TBST trice, protein bands on membranes were visualized using an ultra‐sensitive enhanced chemiluminescent substrate (Thermo Scientific, USA). Photos of the bands were captured by ChemiDocTM MP Imaging System (BIO‐RAD, USA) and band intensity were determined with Bio‐Rad Image Lab 3.0 software. CD54/ICAM‐1 (E3Q9N) rabbit mAb (Cat#67836), Phospho‐NF‐κB p65 (Ser536) (93H1) rabbit mAb (Cat#3033), NF‐κB p65 (D14E12) rabbit mAb (Cat#4595), Phospho‐IκBα (Ser32) rabbit mAb (Cat#2859), IκBα mouse mAb (Cat#4814), Phospho‐IKKα/β (Ser176/180) rabbit mAb (Cat#2697), IKKα (D3W6N) rabbit mAb (Cat#61294), IKKβ rabbit mAb (Cat#8943), phospho‐eNOS (Ser1177) rabbit mAb (Cat#9570), eNOS rabbit mAb (Cat#32027), phospho‐AMPKα (Thr172) rabbit mAb (Cat#2535), AMPKα rabbit mAb (Cat#2532) and β‐Actin (13E5) rabbit mAb (Cat#4970) were all purchased from Cell Signalling Technology (Beverly, MA). VCAM‐1 Antibody (Cat#sc‐13,160) was obtained from Santa Cruz Biotechnology (Texas, USA). ZO‐1 antibody (Cat#61–7300) and Claudin‐5 antibody (Cat#35–2500) were bought from Invitrogen (Carlsbad, CA, USA). Anti‐Occludin antibody (ERP8208) (Cat#ab167161) was purchased from Abcam (Cambridge, UK).

### 2.4 Inflammatory cytokines measurement

The levels of inflammatory cytokines, including interleukin (IL)‐6 and tumour necrosis factor alpha (TNF‐α) in the collected culture medium were measured by the corresponding ELISA kits (Mlbio, China; Cat#ml063160 and Cat#ml002095) in accordance with the kit protocols.

### 2.5 Immunofluorescence staining

The confluent bEnd.3 cells (3 × 10^5^ cells/confocal dish) were pre‐treated with 3,3′,4,5′‐TMS or 3,4′,5‐TMS (5 μM) for 24 h, and then stimulated with OGD for another 2 h. The cells were washed thrice by PBS, fixed with 4% paraformaldehyde (PFA; Beyotime Biotechnology; Cat#P0099) at room temperature for 20 min, and permeabilized with PBS containing 0.1% Triton X‐100 (Sigma Aldrich; Cat#T8787) for 10 min on ice. Following 3‐h blocking by 3% BSA at room temperature for 1 h, the cells were incubated with the primary ZO‐1 antibody (1:500) diluted by 3% BSA at 4°C overnight. Afterwards, the cells were washed by PBS for 10 min followed by incubation with Alexa Fluor 488‐labelled secondary antibody (1:100; Beyotime Biotechnology; Cat#A0423) for another 1 h at 37°C. 2‐(4‐amidinophenyl)‐6‐indolecarbamidine dihydrochloride (DAPI; Beyotime Biotechnology; Cat#C1006) was used to stain the cell nuclei before capturing cell images by Leica‐DMi8 Inverted fluorescence microscope (Leica, Germany).

### 2.6 Measurement of intracellular ROS

Dihydroethidium (DHE; Invitrogen; Cat#D11347) and 5‐(and‐6)‐chloromethyl‐2′,7′‐dichlorodihydrofluorescein diacetate acetyl ester (CM‐H_2_DCFDA; Invitrogen; Cat#C6827) were applied to detect the levels of intracellular superoxide radical (O_2_^•‐^) and hydrogen peroxide (H_2_O_2_) respectively according to the manufacturers' instructions. The bEnd.3 cells (3 × 10^5^ cells/well, 500 μL medium/well in 24‐well plate) were cultured until proper confluence. The cells were exposed to the aforementioned resveratrol derivative and OGD stimulation. After washing with PBS thrice, the cells were incubated with DHE or CM‐H_2_DCFDA at final concentration of 5 μM diluted by normal physiological saline solution (NPSS) at 37°C in the dark for 30 min. After washing thrice, the fluorescence intensity of DHE or CM‐H_2_DCFDA in the cells was detected with Leica‐DMi8 Inverted fluorescent microscope and the integrated optical density (IOD) on cells was analysed with the Image‐Pro Plus 6.0 software. The relative mean fluorescence intensity was quantified and compared among groups.

### 2.7 Permeability assay

The BBB permeability was measured by TRITC‐Dextran leakage assay. The bEnd.3 cells at a density of 5 × 10^4^ cells/well were cultured in the upper chamber of 24‐well transwell plates with 0.4 μm microporous membrane (Labselect, China) for 72 h until full confluence. The cells of treatment groups were pre‐treated with either 3,3′,4,5′‐TMS or 3,4′,5‐TMS (1 and 5 μM) for the last 24 h. The DMEM and glucose‐free DMEM media supplemented with 2 mg/mL TRITC‐Dextran were applied to the upper champers for Control and OGD groups respectively before OGD stimulation. Meanwhile, the respective media were added into the lower chambers. For treatment groups, the glucose‐free media in upper chambers also contained 3,3′,4,5′‐TMS or 3,4′,5‐TMS (1 and 5 μM) besides TRITC‐Dextran. After 2‐h OGD injury, 50 μL medium from both upper and lower chambers of each well was collected and the fluorescence intensity of TRITC‐Dextran was then measured (excitation: 550 nm; emission: 572 nm) by SpectraMax M5 microplate reader. According to the reported formula as following, permeability coeffeicient (*P*_dextran_) was calculated.^1^

$$P_{\text{dextran}}=\left({\mathrm{RFU}}_{\text{lower}}/{\mathrm{RFU}}_{\text{upper}}\right)\times V\times \left(1/t\right)\times \left(1/A\right).$$

RFU: relative fluorescent unit; *V*: volume of bottom well (μL); *t*: incubation time of TRITC‐Dextran (h); *A*: surface area of cell monolayer (cm^2^).

### 2.8 Statistical analysis

All results were presented as means plus standard error of mean (SEM) from three independent experiments. Statistical analysis was calculated by GraphPad Prism software (GraphPad Software, USA). One‐way analysis of variance (ANOVA) and Tukey's post hoc test were performed for comparisons among groups. The *p* < 0.05 was defined as statistically significant.

## 3 RESULTS

### 3.1 Attenuating cell death caused by OGD injury

**FIGURE 2:**
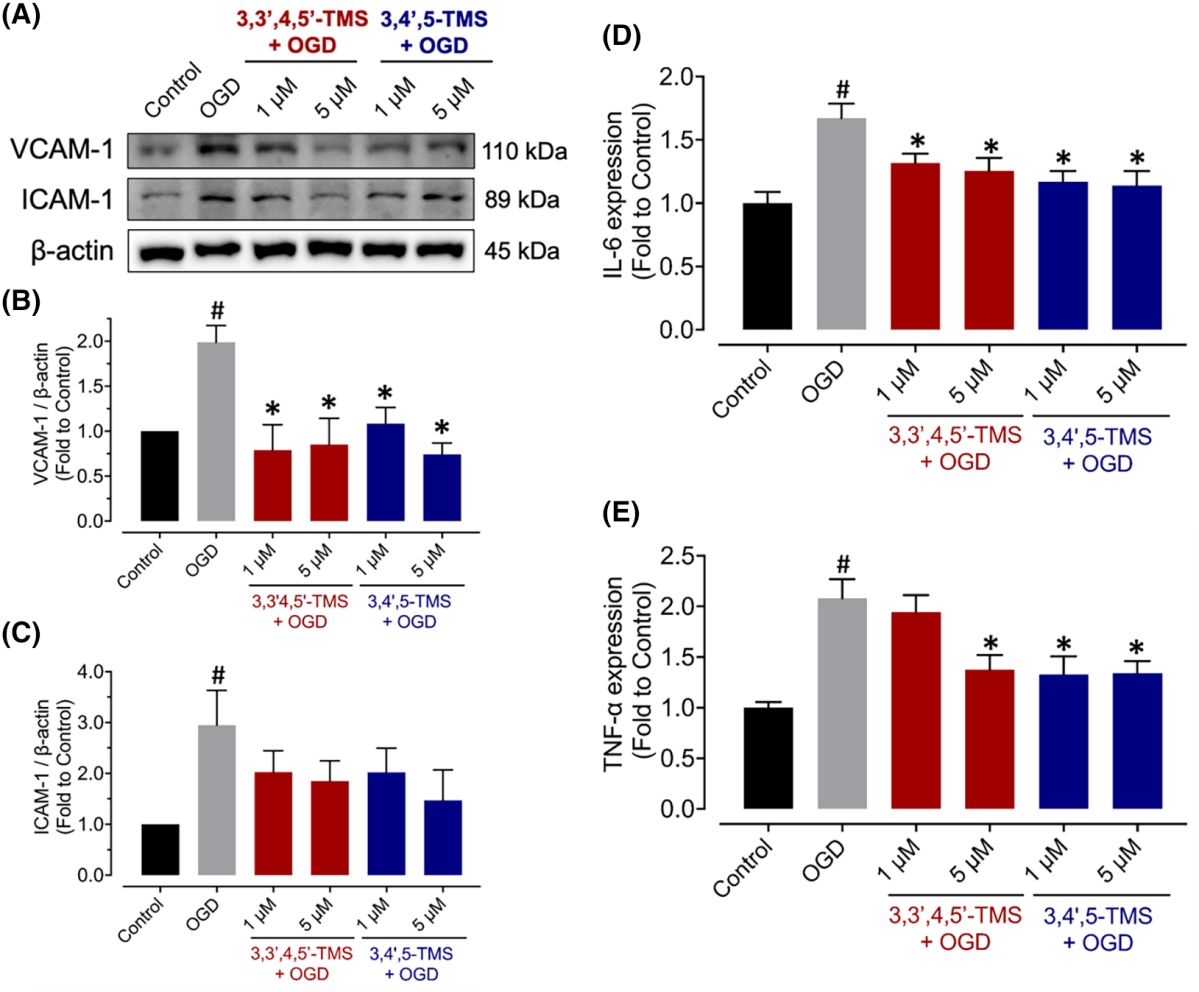
Effects on expressions of inflammatory cytokines in bEnd.3 cells induced by OGD injury. (A) Representative western blots and (B, C) analysed densitometric data for the protein expressions of VCAM‐1 and ICAM‐1 in cells pre‐treated with 3,3′,4,5′‐TMS or 3,4′,5‐TMS at 1 and 5 μM for 24 h and stimulated by 2‐h OGD with normalization with β‐Actin. (D, E) IL‐6 and TNF‐α levels in the cell culture supernatants. ^#^ (vs. Control) and * (vs. OGD) indicated *p* < 0.05 (*n* = 3).

We first evaluated the effects of two stilbenes, 3,3′,4,5′‐TMS and 3,4′,5‐TMS at different concentrations on the cell viability in bEnd.3 cells via MTT assay. The results indicated that both two derivatives could enhance the cell proliferation to some extent but not statistically significant (Figure 1B). Additionally, we also evaluated the cell viability under OGD stimulation. As shown in Figures 1C, 2‐hour OGD decreased cell viability, indicating the cytotoxicity of OGD, while pre‐treatment of 3,3′,4,5′‐TMS or 3,4′,5‐TMS reversed the situation at concentration range of 0.5–5 μM. However, incubation with a concentration of 10 μM of 3,3′,4,5′‐TMS or 3,4′,5‐TMS had no effect on cell viability of OGD‐induced cells. Therefore, we planned to investigate further effects of these derivatives at 1 and 5 μM on OGD‐induced disorders in bEnd.3 cells.

### 3.2 Suppressing OGD‐induced proinflammatory responses and activation of NF‐κB signalling

To explore the effects of both compounds on inflammatory response induced by OGD injury, protein expression levels of VCAM‐1 and ICAM‐1, were measured by western blot assay (Figure 2A–C). 3,3′,4,5′‐TMS and 3,4′,5‐TMS suppressed the elevated expression of VCAM‐1 at 1 and 5 μM but no significant effects were observed on ICAM‐1. Moreover, the release of proinflammatory cytokines including IL‐6 and TNF‐α in the culture supernatants of bEnd.3 cells were induced by OGD injury, and such increases were reduced by pre‐treatment of 3,3′,4,5′‐TMS or 3,4′,5‐TMS (Figure 2D,E). These data indicated that treatment of these two resveratrol derivatives could suppress OGD‐induced inflammatory responses.

**FIGURE 3:**
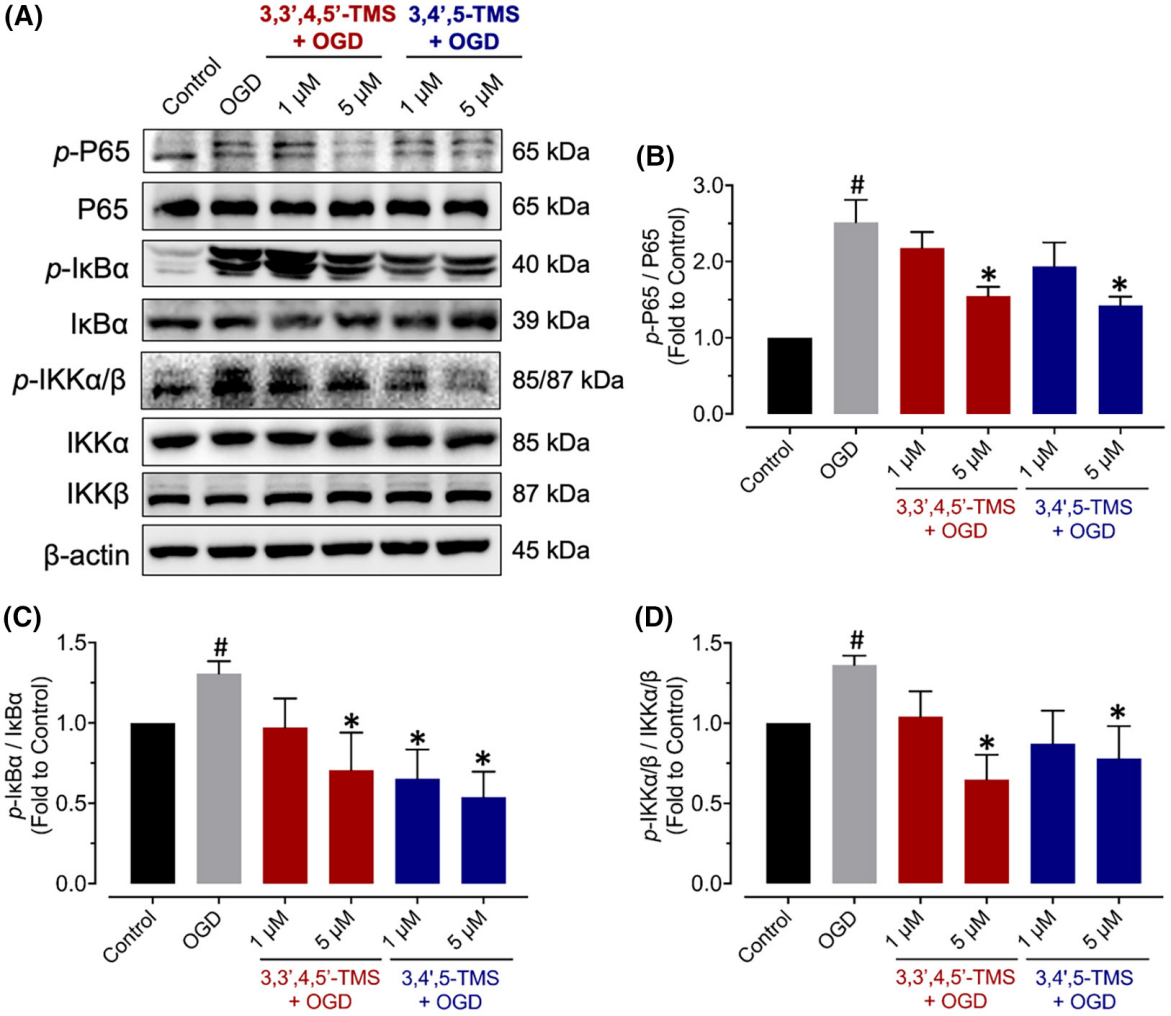
Effects on NF‐κB signalling pathway in bEnd.3 cells after OGD stimulation. (A) Illustrative blots and (B–D) summarized data for phosphorylation of NF‐κB p65 at Ser536, IκBα at Ser32 and IKKα/β at Ser176/180 in cells pre‐treated with 3,3′,4,5′‐TMS or 3,4′,5‐TMS at 1 and 5 μM for 24 h followed by 2‐h OGD normalized with the corresponding total protein. ^#^ (vs. Control) and * (vs. OGD) indicated *p* < 0.05 (*n* = 3).

To elucidate the potential mechanism by which resveratrol derivatives inhibit inflammation in OGD‐stimulated bEnd.3 cells, we measured protein expression and phosphorylation levels on the NF‐κB signalling pathway including NF‐κB p65, IκBα and IKKα/β. As shown in Figure 3, treatment with 3,3′,4,5′‐TMS or 3,4′,5‐TMS at 5 μM attenuated the increased phosphorylation levels of NF‐κB p65 at Ser536, IκBα at Ser32 and IKKα/β at Ser176/180 whereas the lower concentration at 1 μM showed minor effects.

### 3.3 Mitigating ROS generation in bEnd.3 cells upon OGD injury

**FIGURE 4:**
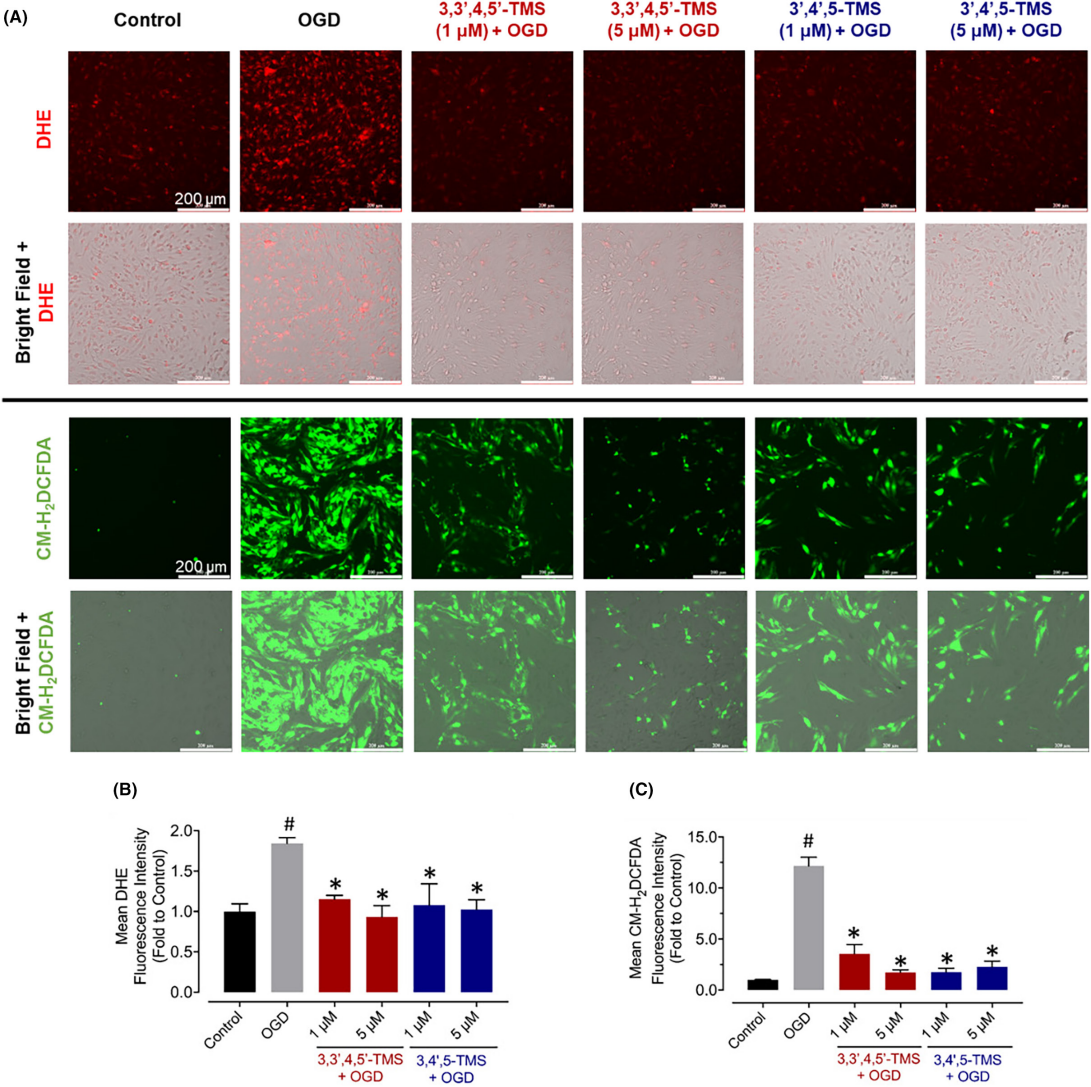
Effects on OGD‐induced ROS in bEnd.3 cells. (A) Representative fluorescence and merged images with bright field, and (B, C) summarized data of fluorescence intensity compared with control group for ROS levels stained by DHE and CM‐H_2_DCFDA in cells exposed to resveratrol derivative and OGD (scale bar = 200 μm). ^#^ (vs. Control) and * (vs. OGD) indicated *p* < 0.05 (*n* = 3).

The intracellular ROS levels including superoxide radical (O_2_^•‐^) and (H_2_O_2_) were detected by two widely used fluorescent probes, DHE and CM‐H_2_DCFDA, respectively. As shown in Figure 4, OGD stimulation caused a remarkable elevation of fluorescence intensity, indicating the accumulation of intracellular ROS. Both 3,3′,4,5′‐TMS and 3,4′,5‐TMS remarkably alleviated ROS generation in bEnd.3 cells under OGD injury.

### 3.4 Restoring endothelial function damaged by OGD

**FIGURE 5:**
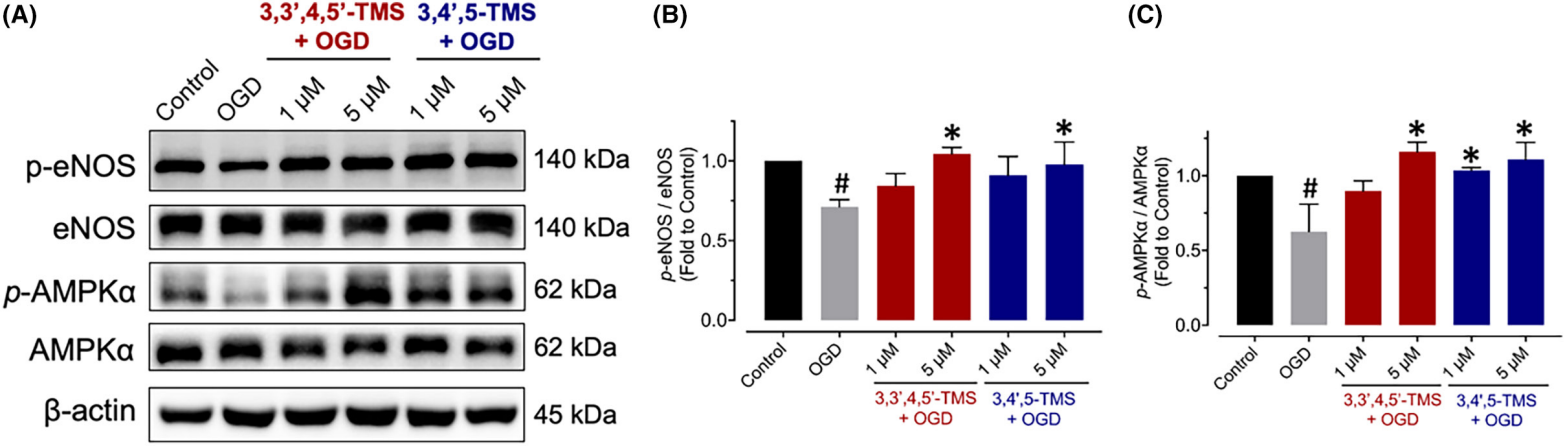
Effects on endothelial dysfunction induced by OGD stimulation. (A) Demonstrative blots and (B, C) analysed results for phosphorylation of eNOS at Ser1177 and AMPKα at Thr172 in cells pre‐treated with 3,3′,4,5′‐TMS or 3,4′,5‐TMS at 1 and 5 μM for 24 h and stimulated by 2‐h OGD as compared to the corresponding total protein. ^#^ (vs. Control) and * (vs. OGD) indicated *p* < 0.05 (*n* = 3).

The enzyme eNOS plays a vital role in maintaining the haemostasis of vascular endothelium. Upon OGD stimulation, phosphorylation of AMPKα at Thr172 and eNOS at Ser1177 was noticeably reduced, and treatments of 3,3′,4,5′‐TMS and 3,4′,5‐TMS significantly enhanced the phosphorylation of these proteins (Figure 5).

### 3.5 Alleviating endothelial barrier injuries through upregulating TJ proteins and rescuing barrier leakage

**FIGURE 6:**
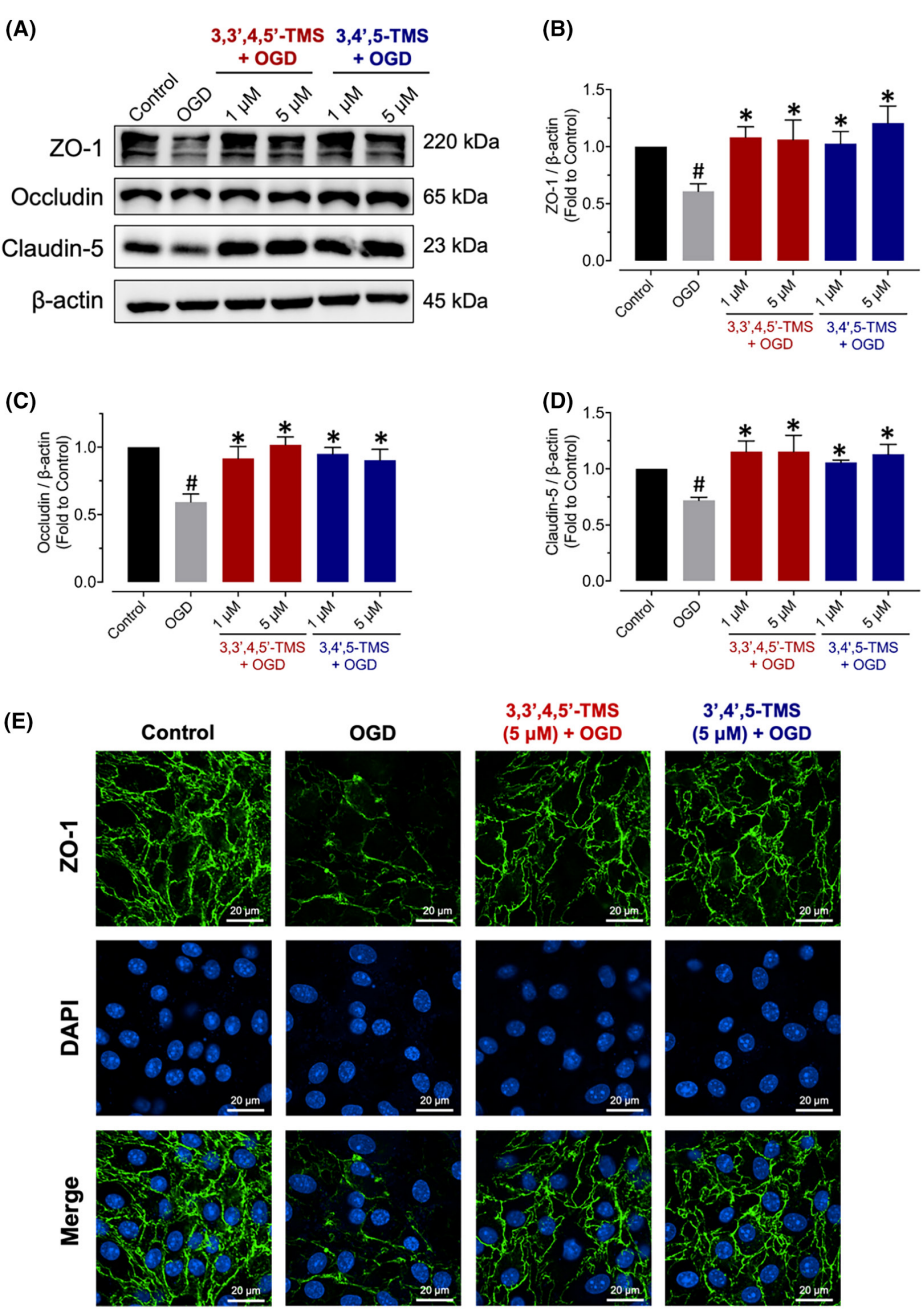
Effects on expressions of tight junction proteins in bEnd.3 cells after OGD injury. (A) Typical western blots and (B–D) graphical data for ZO‐1, occludin, and claudin‐5 in cells pre‐treated with stilbenes at 1 and 5 μM for 24 h followed by 2‐h OGD after normalization to β‐Actin. (E) Representative images of immunofluorescence staining for expression of ZO‐1 (scale bar = 20 μm). ^#^ (vs. Control) and * (vs. OGD) indicated *p* < 0.05 (*n* = 3).

**FIGURE 7:**
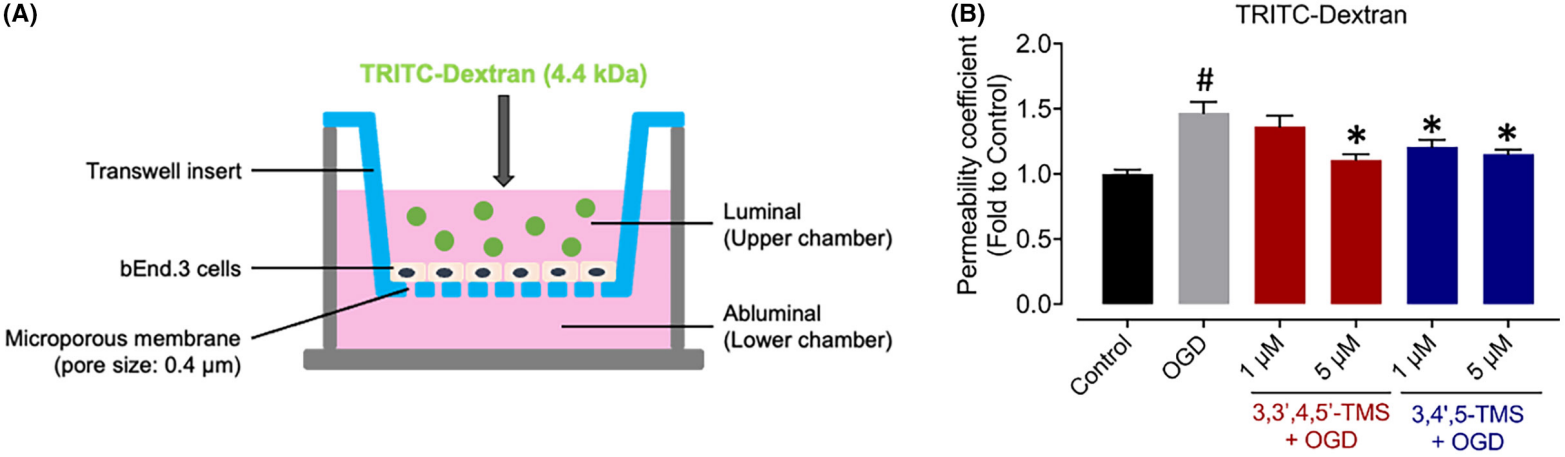
Effects on permeability of bEnd.3 cells after OGD injury. (A) Schematic diagram of transwell insert for measurement of TRITC‐Dextran leakage. (B) Relative permeability coefficient of bEnd.3 cells. The means values ± SEM were from three independent experiments. The value of *p* < 0.05 was marked with ^#^ (vs. Control) and * (vs. OGD).

To evaluate the effects of 3,3′,4,5′‐TMS and 3,4′,5‐TMS on protecting the endothelial barrier against OGD injury, expression levels of three important tight junction (TJ) proteins including ZO‐1, occludin and claudin‐5 were determined by western blot. The stimulation of OGD decreased levels of these TJ proteins while treatment of 3,3′,4,5′‐TMS and 3,4′,5‐TMS effectively ameliorated the endothelial damages by upregulating TJ protein expression (Figure 6A–D). In line with this, immunofluorescence staining showed similar expression levels of ZO‐1 in Figure 6E. Results of permeability assay with TRITC‐Dextran also indicated the barrier leakage after OGD stimulation, which was relieved by 3,3′,4,5′‐TMS and 3,4′,5‐TMS (Figure 7).

## 4 DISCUSSION

In this study, OGD in vitro model was employed to investigate the potential neuroprotective effect and possible underlying mechanisms of two methoxylated derivatives of resveratrol, 3,3′,4,5′‐TMS and 3,4′,5‐TMS. Our results suggested that pre‐treatment of either compound exhibited potential protective activities in BMECs against ischemic stroke in vitro.

Ischemic stroke is the major subtype of cerebral stroke, resulting from the transient or permanent occlusion of cerebral blood vessels. It is a heterogeneous and complicated disorder as a result of multiple factors like age, sex, genetic background, severity and duration of ischemic, localization of infract and physical conditions.^23^ Therefore, experimental models are hard to precisely mimic the reality of ischemic stroke but are still essential for study the mechanisms and screening for potential therapeutic strategies. The OGD stimulation is the most frequently employed in the BMECs as in vitro model and is suitable for high‐throughput research to find potentially neuroprotective pharmaceuticals.^22^, ^24^ To confirm a proper experimental condition, we firstly compared the effects of OGD stimulation from 1 to 4 h (Figure S1) and found that the cell death increased after 2‐h exposure to OGD, and the expression levels of TJ proteins decreased notably. Therefore, we chose 2‐h OGD as the condition for in vitro model of ischemic stroke. As shown in our results, exposure to OGD for 2 h significantly decreased the cell viability while pre‐treatment of 3,3′,4,5′‐TMS and 3,4′,5‐TMS (0.5, 1, and 5 μM) could alleviate the damage. Of note, 3,3′,4,5′‐TMS and 3,4′,5‐TMS at 10 μM did not reduce cell viability in normal BMECs but had no effect on cell viability of OGD‐stimulated BMECs, suggesting possible toxicity of these two compounds at high concentration, particularly in deteriorated cells. Thus, we chose to further explore the anti‐inflammatory and antioxidants effects of 3,3′,4,5′‐TMS and 3,4′,5‐TMS at the safe concentrations, 1 and 5 μM. It is worthwhile to investigate whether the protective effects of the compounds involve inhibition of apoptosis pathways in BMECs under OGD conditions in the future.

Both transient and permanent ischemia can lead to endothelial dysfunction by direct impacts on the ECs or indirect impacts on other NVU components which are regulated by endothelium.^25^ It has been widely accepted that both innate and adaptive immunity take part in pathology of ischemic stroke and various immune cells can react to ischemic injuries, restore the homeostasis, and assist in repair in different manners.^26^ During the acute phase, the immune responses seem to be damaging. Activated microglia, one kind of component in NVU, are the first cells moving to injured site and then release proinflammatory cytokines, ROS, chemokines, prostaglandins, as well as nitric oxide (NO), making more circulating leukocytes can be recruited to promote the neuroinflammation.^27^, ^28^ In the bEnd.3 cells exposed to OGD injury, we noticed the remarkably elevated generations of proinflammatory cytokines including IL‐6 and TNF‐α, as well as intracellular ROS. DHE and CM‐H_2_DCFDA, two commonly used probes for superoxide radical (O_2_^•‐^) and (H_2_O_2_), were applied in this study to measure the intracellular ROS generation. From the results of relative mean fluorescence intensity of staining, 3,3′,4,5′‐TMS and 3,4′,5‐TMS exhibited potency to alleviate the elevated production of ROS induced by OGD stimulation. Pre‐treatment of these two derivatives attenuated such inflammatory response and associated oxidative stress, which was in consistence with previous study about their anti‐inflammatory and antioxidant effects.^21^ However, it is important to note that the inflammatory response is a complex process. In addition to IL‐6 and TNF‐α, measuring other inflammatory mediators such as IL‐1β, IL‐8 or MCP‐1 in the future could provide a more comprehensive understanding of the effects of the two compounds on inflammatory reponses in BMEMs under OGD conditions.

Resveratrol was reported to protect the BMECs against neuroinflammation and oxidative stress under OGD stimulation by several mechanisms including decreasing expression levels of VCAM‐1 and ICAM‐1, inhibiting NF‐κB signalling pathway, and activating AMPK signalling pathway.^11^, ^13^, ^14^ In response to the release of proinflammatory agents, BMECs upregulate the expression levels of VCAM‐1 and ICAM‐1, which subsequently induce transmigration of monocytes and lymphocytes, respectively.^4^, ^29^, ^30^ Consistent with previous studies, we found that both VCAM‐1 and ICAM‐1 were enhanced by OGD induction in bEnd.3 cells, but the treatment of 3,3′,4,5′‐TMS and 3,4′,5‐TMS could only significantly alleviate the expression level of VCAM‐1. NF‐κB, expressed in several cells of central nervous system, is crucial for activation and modulation of neuroinflammation.^31^ The activation of NF‐κB signalling pathway is also regarded as one major cause of endothelial barrier breakdown.^7^, ^32^, ^33^ We measured the protein expression levels of major members of NF‐κB signalling pathway including phosphorylation and total expressions of NF‐κB P65, IκBα and IKKα/β. Two‐hour OGD stimulation activated NF‐κB signalling pathway while pre‐treatment of 3,3′,4,5′‐TMS and 3,4′,5‐TMS at 5 μM for 24 h suppressed this pathway. AMPK, a member of serine/threonine (Ser/Thr) kinase family, serves as an important cellular energy sensor and sustains cerebral metabolic homeostasis through restraining ATP‐consuming processes and promoting catabolic pathways.^34^ Ischemic stroke leading to insufficient supplies of oxygen, glucose and other nutrients, contributes to activation of AMPK. Emerging evidence indicates that protective function of AMPK against ischemic stroke acts through modulating many mechanisms including mitochondrial dysfunction, oxidative stress, apoptosis, autophagy, neuroinflammation and glutamate excitotoxicity.^35^, ^36^, ^37^, ^38^ Since resveratrol is well‐known as an effective AMPK activator and our previous study revealed the vaso‐protective effect of these two derivatives through activation of sirtuin 1 (SIRT1) and AMPK/eNOS pathway, we also detected the phosphorylation level of AMPKα in bEnd.3 cells exposed to OGD.^20^ The results showed these two derivatives enhanced AMPK phosphorylation under OGD injury. Additionally, activated AMPK phosphorylates multiple targets including eNOS, which produces the most important endothelium‐derived relaxing factor (EDRF), NO in maintaining vascular homeostasis.^39^ Our results indicated that OGD stimulation suppressed AMPK/eNOS pathway which was restored by pre‐treatment of 3,3′,4,5′‐TMS and 3,4′,5‐TMS.

BBB is an essential and vital physical and biochemical barrier occurring at the brain microvascular endothelium to control the homeostasis of central nervous system and protect brain tissue against toxic substances.^40^ Endothelial cells, along with other components of NVU, modulate the BBB function. During ischemic stroke, oxygen and nutrient deprivation results in BBB dysfunction through different mechanisms including TJs disruption, abnormal passage of immune cells into the central nervous system from peripheral blood, altered transport mechanisms and dysfunction of components of NVU.^41^ All of these abnormalities contribute to neuroinflammation, oxidative stress, vasogenic edema, impaired water and ion homeostasis, accumulation of toxic substances, BBB disruption and so on.^41^, ^42^ Endothelial cells in CNS, different from those in other regions of our body, exhibit highly specialized TJs, blocking the space between adjacent cells to form the BBB with less transcytosis from the vessel lumen to the brain parenchyma.^43^ Therefore, the TJs among BMECs are of great importance for BBB permeability and integrity. TJ proteins generally include transmembrane proteins (occludin and claudin) and cytoplasmic accessory proteins (zonula‐occludens).^4^, ^44^ Therefore, we detected the expression levels of three most important TJ proteins, ZO‐1, occludin and claudin‐5, under OGD condition and all found an obvious decrease in bEnd.3 cells. Meanwhile, pre‐treatment of 3,3′,4,5′‐TMS and 3,4′,5‐TMS notably mitigated the reduction of TJ protein expression. The immunofluorescence staining results of ZO‐1 also suggested the unclear boundaries and tight junction disruption induced by OGD injury which was ameliorated by 3,3′,4,5′‐TMS and 3,4′,5‐TMS. To better demonstrate their protective effect against barrier disruption, we evaluated the TRITC‐Dextran leakage. After pre‐treatment, the increased monolayer permeability was attenuated significantly.

During the past few decades, numerous effective natural compounds and their derivatives have emerged and are revealed to exhibit promising neuroprotective properties.^2^, ^45^, ^46^, ^47^, ^48^, ^49^ Further investigation is essential to verify the effects and figure out clear mechanisms, so that more potent therapeutic compounds can be revealed for better clinic treatment. In the present study, we showed that two derivatives of resveratrol, 3,3′,4,5′‐TMS and 3,4′,5‐TMS, protect BMECs from OGD injury in vitro, revealing their potential protective effects against ischemic stroke; nevertheless, in vivo effects still need to be investigated and confirmed in the future.

## 5 CONCLUSION

To sum up, our results firstly provide evidence that two methoxylated derivatives of resveratrol, 3,3′,4,5′‐TMS and 3,4′,5‐TMS, attenuate OGD damage and BBB disruption in bEnd.3 cells in vitro. Both compounds alleviate cell death, inflammatory responses, and oxidative stress caused by OGD and also mitigated endothelial barrier injuries through ameliorating the dysfunction of tight junction proteins and restoring endothelial function.

## AUTHOR CONTRIBUTIONS

**Chunxiu Zhou:** Data curation (equal); formal analysis (equal); investigation (equal); methodology (equal); writing – original draft (equal). **Yan Zhou:** Data curation (equal); formal analysis (equal); investigation (equal); methodology (equal); writing – original draft (equal). **Chi Teng Vong:** Investigation (equal); writing – review and editing (equal). **Haroon Khan:** Conceptualization (equal); writing – review and editing (equal). **Wai San Cheang:** Conceptualization (lead); funding acquisition (lead); supervision (lead); writing – review and editing (lead).

## CONFLICT OF INTEREST STATEMENT

The authors declared no conflict of interest.

## Supporting information


Figure S1:

## Data Availability

The data that support the findings of this study are available from the corresponding author upon reasonable request.
