# Supplementary material for: 3,3′,4,5′‐Tetramethoxy‐trans‐stilbene and 3,4′,5‐trimethoxy‐trans‐stilbene prevent oxygen–glucose deprivation‐induced injury in brain endothelial cell
Source: J Cell Mol Med. 2024 Aug 17;28(16):e70008. doi: 10.1111/jcmm.70008 (PMC11330235; doi:10.1111/jcmm.70008)
Supplement: Supplementary file 1 — Figure S1: [file JCMM-28-e70008-s001.pdf]

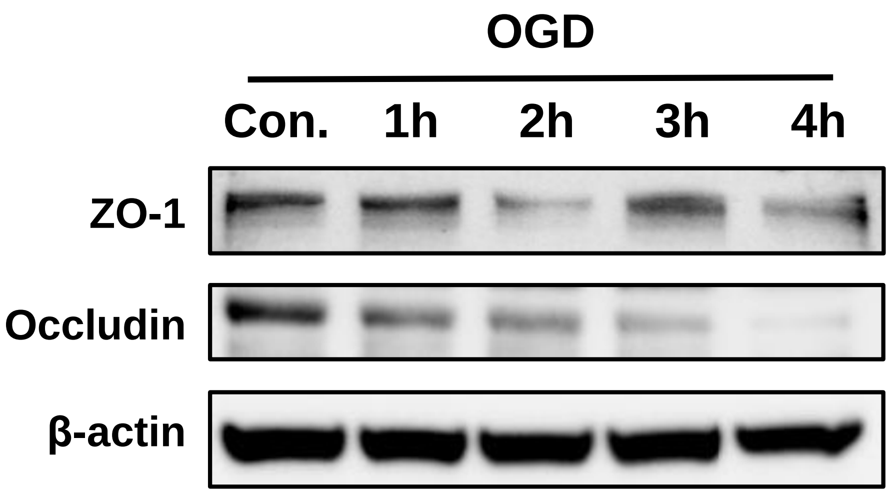

Supplementary Figure 1. Effects of OGD stimulation from 1 to 4 hours on expressions of tight junction proteins, ZO-1 and occluding.
